# Adoption and Appropriateness of mHealth for Weight Management in the Real World: A Qualitative Investigation of Patient Perspectives

**DOI:** 10.2196/29916

**Published:** 2021-12-08

**Authors:** Jessica Y Breland, Khizran Agha, Rakshitha Mohankumar

**Affiliations:** 1 Center for Innovation to Implementation VA Palo Alto Health Care System Menlo Park, CA United States

**Keywords:** mHealth, implementation, adoption, engagement, weight management, obesity, weight loss, mobile health, veterans, barriers

## Abstract

**Background:**

Mobile health (mHealth) interventions for weight management can result in weight loss outcomes comparable to in-person treatments. However, there is little information on implementing these treatments in real-world settings.

**Objective:**

This work aimed to answer two implementation research questions related to mHealth for weight management: (1) what are barriers and facilitators to mHealth adoption (initial use) and engagement (continued use)? and (2) what are patient beliefs about the appropriateness (ie, perceived fit, relevance, or compatibility) of mHealth for weight management?

**Methods:**

We conducted semistructured interviews with patients with obesity at a single facility in an integrated health care system (the Veterans Health Administration). All participants had been referred to a new mHealth program, which included access to a live coach. We performed a rapid qualitative analysis of interviews to identify themes related to the adoption of, engagement with, and appropriateness of mHealth for weight management.

**Results:**

We interviewed 24 veterans, seven of whom used the mHealth program. Almost all participants were ≥45 years of age and two-thirds were White. Rapid analysis identified three themes: (1) coaching both facilitates and prevents mHealth adoption and engagement by promoting accountability but leading to guilt among those not meeting goals; (2) preferences regarding the mode of treatment delivery, usability, and treatment content were barriers to mHealth appropriateness and adoption, including preferences for in-person care and a dislike of self-monitoring; and (3) a single invitation was not sufficient to facilitate adoption of a new mHealth program. Themes were unrelated to participants’ age, race, or ethnicity.

**Conclusions:**

In a study assessing real-world use of mHealth in a group of middle-aged and older adults, we found that—despite free access to mHealth with a live coach—most did not complete the registration process. Our findings suggest that implementing mHealth for weight management requires more than one information session. Findings also suggest that focusing on the coaching relationship and how users’ lives and goals change over time may be an important way to facilitate engagement and improved health. Most participants thought mHealth was appropriate for weight management, with some nevertheless preferring in-person care. Therefore, the best way to guarantee equitable care will be to ensure multiple routes to achieving the same behavioral health goals. Veterans Health Administration patients have the option of using mHealth for weight management, but can also attend group weight management programs or single-session nutrition classes or access fitness facilities. Health care policy does not allow such access for most people in the United States; however, expanded access to behavioral weight management is an important long-term goal to ensure health for all.

## Introduction

Obesity affects roughly 40% of adults in the United States [1] and is associated with many negative outcomes, including social stigma and chronic conditions, such as diabetes and heart disease [2,3]. Although weight management requires long-term, complex behavior change, behavioral weight management programs result in reduced weight, morbidity, and mortality [4-6]. For example, MOVE!, the Veterans Health Administration’s (VHA) flagship weight management program, has been associated with reductions in cardiovascular disease [7] and reductions in diabetes complications [8]. However, while 94% of VHA patients with overweight or obesity are offered weight management programs, only 10% use them [9]. Research outside the VHA in the United States does not typically focus on initial engagement in weight management programs because it is difficult to define the denominator of the target population. However, existing work outside Veterans Affairs (VA) also suggests low engagement rates [10-14].

Mobile health (mHealth) interventions for weight management can result in weight loss outcomes comparable to in-person treatments, although results are mixed [15-17]. For example, Track is an mHealth intervention for adult patients with obesity and related comorbidities (eg, diabetes, hypertension) [18]. It provides app-based self-monitoring in addition to dietician-delivered counseling calls with tailored feedback. In a 12-month effectiveness randomized controlled trial at a community health center, participants randomized to Track were more likely to lose at least 5% of their baseline weight. Given the ubiquity of smartphones [19], increasing access to mHealth for weight management could increase weight management program use and improve patient health. However, there is relatively little information on implementing mHealth for weight management in routine practice settings, particularly among older adults and with regard to patient-level factors [20].

In this work, we performed a qualitative analysis of patient interviews to answer two research questions about implementation outcomes among a sample of primary care patients with obesity: (1) what are barriers and facilitators to mHealth adoption and engagement? and (2) what are patient beliefs about the appropriateness of mHealth for weight management? We conducted this work at the VA Palo Alto Health Care System, which had recently implemented an evidence-based [21] commercial smartphone app designed to facilitate weight management and other health behaviors. Starting in March 2019, VA Palo Alto patients were offered access to this app for at least 6 months. The mHealth program includes app-based content (eg, self-monitoring, information modules, and exercises) and a live coach to facilitate goal setting and attainment. Depending on patient preference, the content on the app can be used alone or with coach support. There is also more formal coach-supported content (eg, sessions based on the Diabetes Prevention Program). As noted by Hermes and colleagues [20], obtaining patient-level information on adoption of, engagement with, and appropriateness of mHealth interventions is especially important for implementing patient-facing mHealth. VHA was an important setting for this work as VHA patients represent a population not typically studied in mHealth contexts (eg, older adults [22]).

## Methods

### Recruitment and Interviews

Starting in March 2019, the VA Palo Alto Health Care System offered primary care patients with obesity access to an mHealth program for weight management (Vida [21]). The mHealth program was tailored for VHA patients—for example, there was an effort to hire coaches with firsthand military experience or military experience via family members, or those who worked in the military as civilians. Coaches also received online training in military culture via VHA’s Talent Management System.

The program was advertised to and by primary care clinicians, behavioral health staff, and weight management clinicians. It was also advertised at the main hospital’s weekly farmers market, and through flyers and social media. In consultation with the Public Affairs Office, ads and images were selected to represent a diverse population with regard to age, sex, and ethnicity. After learning about the mHealth program in one of the aforementioned ways, patients had to contact VA Palo Alto staff, express interest, and meet minimal criteria (ie, access to a smartphone with internet, no uncontrolled mental or physical health conditions). Patients were then given an access code they could use to download the app and begin the mHealth program.

Between February 2020 and October 2020, we used administrative data to obtain a randomly chosen list of 77 potential participants who were VA Palo Alto adult primary care patients with obesity (ie, body mass index ≥30) who had been given an mHealth access code. We excluded 36 patients from this list who no longer had a BMI ≥30, or who had a hospitalization or suicide attempt in the past 30 days, cognitive impairment, and/or a psychotic disorder diagnosis. We mailed opt-out letters to the remaining 41 patients, calling potential participants who did not opt out or otherwise contact study staff. Interested and eligible patients completed the consent process and a one-time interview over the phone (n=24 for a response rate of 59%). We mailed opt-out letters in February, August, and October 2020. We completed interviews in February, March, June, September, October, and November 2020. Therefore, data collection was complete by November 2020. All participants who completed the consent process also completed the interview. Interviews lasted roughly 45-60 minutes. Detailed, typewritten notes were taken during interviews. COVID-19–related technology problems prevented staff from recording all interviews. However, roughly half of the interviews were digitally audio-recorded (n=11). In most cases, each interview consisted of one staff member and one participant. The semistructured interview guide covered beliefs about weight and weight management and was part of a larger study on that topic. Questions most relevant to the present work are in Table 1 (full guide available upon request). We paid participants $50.

**Table 1:** Most relevant interview questions and prompts.

Question	Relevant prompts
Have you ever tried to lose weight?	N/A^a^
Why did you try to lose weight?	N/A
How have you tried to lose weight?	How/where did you learn about/use Vida? What made it easy to learn about Vida? What made it difficult to learn about Vida?
What got in the way of you trying to lose weight?	What got in the way of using Vida? Would tailored information have changed your experience with Vida? Did the coach and examples seem relevant to you? What would have helped you use Vida? What do you think of the idea of an app with a live coach to help with weight management?
What helped you try to lose weight?	What helped you use Vida? How did the coach affect your experiences with Vida?

^a^N/A: not applicable.

### Analysis

We used rapid qualitative analysis methods informed by Neal and colleagues [23] and Nevedal and colleagues [24] to answer the research questions: (1) what are barriers and facilitators to mHealth adoption (initial use) and engagement (continued use, ie, 2 or more uses)? and (2) what are patient beliefs about the appropriateness (ie, perceived fit, relevance, or compatibility [20]) of mHealth for weight management? Rapid analysis has been shown to be faster than and as effective as other forms of qualitative analysis for relatively straightforward research questions such as ours [24,25].

During and after each interview, we completed a matrix in Microsoft Excel (Microsoft Corp) to identify key information about participants’ mHealth experiences. The matrix had a row for each participant. Columns included deductive codes related to our primary research questions: whether participants used Vida, mHealth barriers, and mHealth facilitators. There were societal barriers and facilitators columns to note factors related to sociodemographic information and/or discrimination. There was also an “other notes” column, where researchers could document information that did not fit into the aforementioned columns. After all other information was entered into the matrix, we added columns for participants’ gender, age, and race/ethnicity to help us identify whether there were patterns across those demographic groups. Analysts also wrote a memo for each participant describing the entire interview.

After all interviews were complete, JYB and KA separately performed inductive analysis by reviewing each barriers and facilitators column and looking for themes—that is, repetition of topics, salience of topics to participants, and negative cases (cases that were different or unique compared to other people). They used the same process across rows to identify possible themes within participants, finding none. The authors then met to review themes and resolve discrepancies (there were few), following which the two authors separately reviewed interview memos to search for additional information on existing themes or information on new themes. After meeting to compare results, noting high agreement and no new themes, the authors settled on a final list of themes and representative quotes from participants. All methods were approved by the Stanford University Institutional Review Board.

## Results

### Overview

We interviewed 24 veterans, 6 of whom were women. Only 2 participants were aged <45 years, and we had roughly equal numbers of participants aged 45-64 years and ≥65 years. Two-thirds of our sample was White; other participants reported their race/ethnicity as Black or African American, Asian, Hispanic, or other. Table 2 provides additional detail. A total of 7 participants reported adopting the mHealth program, 4 of whom were women. For simplicity and to use the language of mHealth, in the sections below we refer to participants who adopted the mHealth program as users and those who did not adopt the mHealth program as nonusers.

**Table 2:** Participant characteristics.

Characteristics			Total		Adopted mHealth intervention		Did not adopt mHealth intervention
**Gender**							
	Woman	6		4		2	
	Man	18		3		15	
**Race/ethnicity**							
	White	16		5		11	
	Black or African American, Asian, Hispanic, and/or other	8		2		6	
Total			24		7		17

We identified three themes related to the adoption of, engagement with, and appropriateness of mHealth, which are described below. Other than noting a greater proportion of women used the mHealth program than men, we identified no themes related to gender, age, or race/ethnicity. Some findings did not fit under the specific themes, but influenced the presentation of themes. First, most participants had tried multiple weight loss methods, including other forms of mHealth. Second, barriers and facilitators to mHealth adoption were similar between users and nonusers, although the latter necessarily described hypothetical reasons. Finally, improving overall health was the main weight management motivator for participants.

### Theme 1: Coaching Both Facilitates and Prevents mHealth Adoption and Engagement

A participant who used the mHealth program described this theme most succinctly: “the coach…is a double-edged sword, people need accountability, but at the same time they are sometimes afraid of accountability.”

Participants said the live coach facilitated adoption and engagement by facilitating accountability. As a user noted, the coach “makes you responsible to answer to someone,” adding that the accountability was “a big part of [my] success, setting goals and expectations helped me a lot.” This was also true for nonusers, with seven of 17 participants who did not use the mHealth program saying they liked the idea of receiving health coaching via mHealth and two expressing a desire to get another the mHealth program access code after the interview. As one participant who did not use the mHealth program said, the coach was appealing because “I always desire to have someone whom I am accountable to, who is knowledgeable and supportive. In the end we are all humans and need support.” Of note, users and nonusers generally did not report a desire to have a coach matched to them solely on demographic characteristics (“I don’t care if they’re male, female, Black, white”). Instead, they focused on the importance of connecting to the coach in at least one way, which they were as likely to describe as being related to hobbies (eg, types of physical activity) versus a demographic characteristic (eg, gender).

At the same time, participants also described how coaches could prevent adoption and engagement. First, feelings of guilt engendered by not meeting goals led participants to stop meeting with the coach and to stop using the app. For some participants, this was also linked to shame, with one participant stating the following:

I didn’t like the feeling of answering to someone when I am not successful, I felt like I wasn’t doing enough, and I used to feel ashamed when I would think about talking to the coach. This made me resistant to using Vida.

For this participant, the feelings applied to doctors as well. For others, the guilt was linked to past health struggles:

I can’t think of any drug addict or person who has a flaw that likes to continuously focus on it. For me it was like, jeez, I have to keep talking about this. Did I do this food? Did I do this activity? And it’s like ah, I didn’t do it today. I would feel bad because I didn’t stick to the plan. I know I’m not disappointing her, but that’s just the way it would go.

Notably, this participant acknowledged that the coach was not judgmental, but that did not alleviate feelings of guilt. Comments from nonusers also suggest a fear of disappointing even a hypothetical coach:

Yes, what I really like about the idea is being accountable to somebody. Being able to talk to someone who can understand your issue and is going to be helpful. I would like my coach to be encouraging, supportive and understanding. [A coach that doesn’t] make a big deal when you miss something [and] instead says, “it’s okay you missed it today it’s not end of the world, start a new day from tomorrow.”

High coach turnover rates also prevented mHealth engagement as it resulted in some participants having multiple coaches. Participants described frustration arising from multiple rapport-building attempts with new coaches, and ultimately disengagement. As one participant said:

[The coach] enhanced the whole experience, I looked forward to talking to her every week, but then I stopped losing weight which frustrated me…and then [I] had to travel and my counselor changed. I didn’t feel like building a relationship with a new person.

### Theme 2: Preferences Regarding the Mode of Treatment Delivery, Usability, and Treatment Content Were Barriers to mHealth Appropriateness and Adoption

While participants said mHealth was generally appropriate for weight management, they also noted that it was not appropriate in all cases. In this way, the mode of treatment delivery served as a barrier for users and nonusers alike. For example, some participants simply preferred in-person treatment. As a nonuser noted, “I like the convenience of phone meetings, but there is a better accountability when you have in person appointments.” Another nonuser suggested connecting patients to peers, for example*,* “through a connected app where you’re connected with somebody else in a different part of the country…and you’re both going to lose weight together. You have a friend and you’re committed with somebody.”

Some users described a preference for in-person treatment, despite being willing to try mHealth. One noted that the mHealth program may not have worked for her because the coach “was on the video thing…she wasn’t in my face, she didn’t know me a whole lot,” adding that “it’s a physical thing, in person, not over the phone. I just didn’t connect with [virtual care].”

Half of participants who adopted the mHealth program thought usability was poor—namely, that entering dietary information was more difficult than other apps they used. Some participants noted they were already using a different kind of mHealth for weight management. With regard to treatment content, several participants said they did not use the mHealth program or did not like using the mHealth program because they did not like monitoring their food. Although, one participant noted that “I can’t say that I enjoy logging my food, but I have to acknowledge that it makes a difference.”

Participants also noted that treatment preferences could change with life circumstances and competing demands. For example, some noted that they learned about the mHealth program toward the end of MOVE! and thought it would serve as way to maintain weight management behaviors once MOVE! ended. However, for at least one participant, by the time MOVE! ended, his daughter’s needs superseded his own goals and prevented mHealth adoption. For a user, her husband’s mental health care took a substantial amount of time, which left her limited time to engage in health behavior change. In addition, as another user noted, one’s own health could prevent engagement in mHealth-related behaviors: “I was swimming for a few months and then I had an open sore and I [couldn’t] get into the pool.”

### Theme 3: A Single Invitation Was Not Sufficient to Facilitate Adoption of a New mHealth Program 

All participants in this study were given an access code for the mHealth program. However, the most common—and therefore primary—barrier to mHealth adoption among participants was not remembering they received an access code, with 12 of 17 nonusers giving this response. Of these 12, two participants remembered seeing ads for the mHealth program, suggesting some familiarity with the program.

## Discussion

### Principal Findings

This is one of the first studies to assess adoption and appropriateness of mHealth for weight management among a group of middle-aged and older adults in a real-world setting. Most participants viewed mHealth as appropriate for weight management. However, despite seeking out and being given free access to an app with a live coach, the majority did not complete the registration process. As a result, the primary barrier to adoption was not remembering they had access, suggesting potential problems with the feasibility of the implementation of this mHealth intervention. For those who adopted the mHealth intervention, barriers to engagement included guilt and shame related to not meeting goals, competing demands of everyday life, and poor usability. Notably, these barriers were not related to the age of participants. Other than women being more likely to use the mHealth program, we did not find noticeable patterns by gender, age, race, or ethnicity.

Perhaps the most novel finding is that coaching was a double-edged sword for participants. Coaching enticed people to adopt the mHealth intervention. However, if they did not consistently achieve their goals, coaching became a barrier to continued engagement. Both guilt (discomfort with one’s actions) and shame (feeling like a person who cannot achieve goals) were forces for disengagement [26]. There is a literature on guilt and shame in relation to health behaviors. For example, Thøgersen-Ntoumani and colleagues found that higher ratings of self-compassion in response to dietary lapses were associated with less guilt and stronger goal perseverance intentions and self-efficacy during weight loss attempts [27]. These results suggest that promoting self-compassion may encourage participation in coaching interventions when guilt and shame may be at play. However, guilt, shame, and self-compassion have not been a focus of the health coaching literature. Our findings suggest this may be an important area of future research. It is possible that mHealth coaching for weight management requires more advanced training to build the rapport necessary to overcome guilt and shame. 

In addition, our findings highlight the importance of acknowledging and working with ambivalence during behavior change interventions. Motivational interviewing, which is a counselling method used to resolve ambivalence, is associated with weight loss in the context of eHealth and telehealth weight loss programs [28]. Although it is a part of Vida coach training, our findings suggest that coaches may need to spend additional time addressing ambivalence to ensure continued engagement. Further, our findings related to guilt, shame, and ambivalence may explain why coaching did not improve outcomes in an mHealth intervention designed to increase physical activity among veterans, even though coaching was front-loaded to increase engagement [29]. These factors should be addressed in future research.

It is also notable that participants generally did not have demographic criteria for coaches. Instead, they focused on a need to connect with the coach across varied affinities. In this study, the mHealth program was adjusted and coaches were trained to account for military culture. Therefore, while not mentioned by participants, it is possible that military-related tailoring was sufficient for participants to feel an affinity to the coach. This is heartening given that the intersectional nature of identity [30] means that it is impossible to demographically match all patients and coaches. At the same time, data from in-person medical settings suggest that demographic matching can influence health outcomes; for example, mortality is lower for Black infants when Black physicians provide care [31]. An important area of future research includes determining whether outcomes are better when coaches are matched on specific characteristics and whether this is more important for historically marginalized populations.

Our results also add to other findings from VHA patients using a web-based weight management program. A qualitative study of women veterans using an online version of the Diabetes Prevention Program had some similar results—the women liked the ability to access intervention materials at any time on the internet, but some did not like self-monitoring [32]. That digital program resulted in higher participation rates than standard VHA weight management programming [33], further highlighting the potential utility of digital health in this context.

Our most actionable finding may be that a single referral to mHealth is insufficient for adoption. Although this finding is related to a relatively simple theme, solutions may be complex. The Expert Recommendations for Implementing Change (ERIC) suggest implementation strategies that might facilitate adoption, including developing and distributing educational materials, obtaining and using patient and family feedback, and using mass media [34]. In the context of mHealth, this could include repeated follow-up contacts via phone, SMS text message–based reminders, and/or marketing materials sent to interested patients more than once. Implementation efforts of other digital health programs in VHA suggest that engaging clinicians and leadership will be especially important [35]. Health care systems could also learn from the private sector while using resources like the Digital Health Checklist to ensure a match between private sector and health care ethical standards [36].

### Limitations

Limitations include a focus on VHA participants. VHA is an integrated health care system that provides primary and specialty physical and mental health care to its patients. People receiving care in stand-alone clinics may have different experiences. In addition, we did not have objective information on whether participants used a specific mHealth program within the app (eg, a program that had a specific number of sessions) as barriers and facilitators could differ across finite versus infinite programs. We also do not have information regarding participants’ views of military-related tailoring. Although the random sample of users invited to participate in this research should have helped account for potential differences between people who used the program earlier versus later, given our small sample and limited information about the denominator of people offered the program, the sample may not be representative.

The primary limitation of this study relates to a primary conclusion—few participants who received an access code to a new mHealth program adopted it. Including fewer users than nonusers in our sample could have led to missed themes related to adoption. However, similarities in descriptions of barriers and facilitators to adoption between users and nonusers ameliorate this concern. Other strengths include our older sample and an analysis based on real-world mHealth use, which are not commonly studied.

### Conclusions

Our findings suggest that implementing an mHealth intervention for weight management in an integrated system primarily serving older adults requires more than one information session. Findings also suggest that focusing future research on the coaching relationship and how users’ lives and goals change over time will be important for facilitating engagement and improved health. Most participants thought the mHealth intervention was appropriate for weight management, with some nevertheless preferring in-person care. Therefore, the best way to ensure equitable care will be to ensure multiple routes to achieving the same behavioral health goals. VHA patients have the option of using mHealth for weight management, but can also attend group weight management programs or single-session nutrition classes, or access fitness facilities. Health care policy does not allow this for most people in the United States; however, expanded access to behavioral weight management programs is an important long-term goal to ensure health for all.

## Abbreviations

ERIC: Expert Recommendations for Implementing Change
mHealth: mobile health
VA: Veterans Affairs
VHA: Veterans Health Administration
